# Different Cytotoxic Effects of Cisplatin on Pancreatic Ductal Adenocarcinoma Cell Lines

**DOI:** 10.3390/ijms252413662

**Published:** 2024-12-20

**Authors:** Antonella Muscella, Luca G. Cossa, Erika Stefàno, Gianluca Rovito, Michele Benedetti, Francesco P. Fanizzi, Santo Marsigliante

**Affiliations:** Dipartimento di Scienze e Tecnologie Biologiche e Ambientali (Di.S.Te.B.A.), Università del Salento, Via Provinciale per Monteroni, 73100 Lecce, Italy; lucagiulio.cossa@unisalento.it (L.G.C.); erika.stefano@unisalento.it (E.S.); gianluca.rovito@unisalento.it (G.R.); michele.benedetti@unisalento.it (M.B.); fp.fanizzi@unisalento.it (F.P.F.); santo.marsigliante@unisalento.it (S.M.)

**Keywords:** cisplatin, pancreatic ductal adenocarcinoma, cytotoxicity, signal transduction, apoptosis

## Abstract

This study examined the response to cisplatin in BxPC-3, Mia-Paca-2, PANC-1, and YAPC pancreatic cancer lines with different genotypic and phenotypic characteristics, and the mechanisms associated with their resistance. BxPC-3 and MIA-PaCa-2 cell lines were the most sensitive to cisplatin, while YAPC and PANC-1 were more resistant. Consistently, in cisplatin-treated BxPC-3 cells, the cleavage patterns of pro-caspase-9, -7, -3, and PARP-1 demonstrated that they were more sensitive than YAPC cells. The autophagic pathway, promoting cisplatin resistance, was active in BxPC-3 cells, as demonstrated by the time-dependent conversion of LC3-I to LC3-II, whereas it was not activated in YAPC cells. In cisplatin-treated BxPC-3 cells, Bcl-2 decreased, while Beclin-1, Atg-3, and Atg-5 increased along with JNK1/2 phosphorylation. Basal levels of phosphorylated ERK1/2 in each cell line were positively correlated with cisplatin IC50 values, and cisplatin caused the activation of ERK1/2 in BxPC-3 and YAPC cells. Furthermore, ERK1/2 pharmacological inactivation increased cisplatin lethality in both BxPC-3 and YAPC cells, suggesting that p-ERK1/2 may be related to cisplatin resistance of PDAC cells. Different mechanisms and strategies are generally required to acquire drug resistance. Here, we partially explain the other response to cisplatin of BxPC-3 and YAPC cell lines by relating it to the role of ERK pathway.

## 1. Introduction

Pancreatic cancer, despite having a relatively modest incidence of new cases, is in fourth place in the ranking of tumors with the highest deaths [1,2], with a high capacity to develop relapses and a marked chemoresistance [3,4,5]. Thus, it remains one of the most aggressive tumors, and patients with pancreatic cancer usually have a poor prognosis [6]. In addition, most patients are diagnosed at an advanced stage of the disease, often due to the lack of specific and easily recognizable symptoms [7]. Unfortunately, it is not possible to intervene surgically on most of the patients with this type of tumor; so, the only therapy provided is chemotherapy, which in most cases represents only a palliative treatment [8]. Therefore, considering the severity of the disease and the limited effectiveness of the treatments currently available, it would be useful to understand the molecular mechanisms that are activated in these cells when treated with cytotoxic molecules. Nowadays, gemcitabine is the first-line therapy for pancreatic cancer, approved by the U.S. Food and Drug Administration [FDA] in 1996. Indeed, gemcitabine causes apoptosis of malignant pancreatic cancer cells [9,10,11]. However, the increasingly common development of gemcitabine resistance during chemotherapy negatively affects the prognosis of pancreatic carcinoma [12]. Intrinsic and acquired factors are involved in gemcitabine resistance. Several of them are related to the transport and metabolism of gemcitabine [13,14] and/or are associated with the tumor microenvironment, among others [15,16].

Recently, platinum-based drugs, such as cisplatin, have been used with slightly better success than gemcitabine, but at the expense of serious side effects and toxicity [17]. The extent of responses varies greatly with both drugs, increasing the need for alternative therapeutic approaches in those cases in which tumor cells became resistant to the therapeutic agents [18]. The response of various PDAC cell lines to cisplatin, including BxPC-3, MIA PaCa-2, PANC-1, and YAPC cells, showed differing results regarding sensitivity and resistance. For instance, one study reported that BxPC-3 and MIA PaCa-2 cells were more sensitive to chemotherapy, while PANC-1 and YAPC cells exhibited higher resistance due to mesenchymal traits and genetic alterations such as KRAS mutations [14,18,19,20].

Other studies have characterized the phenotypic and genotypic differences between these cell lines, highlighting the critical role of the KRAS mutation and other factors in chemoresistance [21,22].

To provide a novel comparative analysis of cisplatin responses across different PDAC cell lines, in this study we used four pancreatic cancer cell lines [BxPC-3, Mia Paca-2, PANC-1, YAPC] with several different genotypic and phenotypic characteristics to examine and evaluate any differences in their response to cisplatin, focusing on the activation of the ERK1/2 pathway and its potential role in enhancing drug resistance. By exploring the interplay between ERK1/2 signaling and autophagy, our study provides new insights into the mechanistic basis of cisplatin resistance in PDAC, which may inform future therapeutic strategies.

## 2. Results

### 2.1. Cytotoxicity of Cisplatin in Pancreatic Tumor Lines

We assessed the cytotoxic potential of cisplatin on the four cell lines examined, stimulating cells with increasing concentrations (0.1–200 µM), to estimate the IC50 value, by the sulforhodamine B (SRB) colorimetric assay. Furthermore, results comparable to those of SRB were obtained when cell numbers were determined directly by cell counting; therefore, we used the SRB assay in the reported combined experiments.

**Figure 1:**
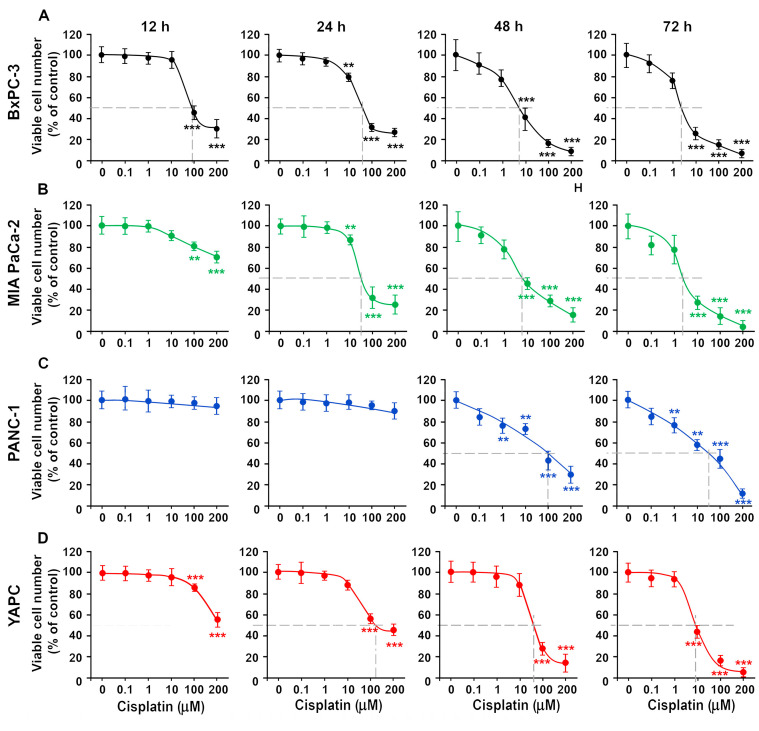
Cytotoxic effects of cisplatin on pancreatic tumor lines. BxPC-3 (**A**), Mia Paca-2 (**B**), PANC-1 (**C**) and YAPC (**D**) cells were treated with different concentration of cisplatin (0.1–200 µM). Cell viability was measured with sulforhodamine B (SRB) colorimetric assay, after 12, 24, 48, or 72 h. Data are the means ± standard deviation (SD) of five independent experiments with eight replicates in each and are presented as percent of control at the corresponding time point, with the control set as 100% ** *p* < 0.01; *** *p* < 0.001 by one-way ANOVA followed by Bonferroni/Dunn post hoc tests. The dashed lines indicate the IC50 values.

Cisplatin (0.1–200 mM) caused a dose- and time-dependent (12–72 h) reduction in cell survival. From the graphs in Figure 1 and Supplementary Table S1, cisplatin has different cytotoxic efficacy in different cell lines. The most sensitive cell lines to the action of cisplatin are the BxPC-3 and the MIA PaCa-2 (The IC50 values indicated below are at 48 h of incubation as these are obtainable in all cell lines only after this time: IC50 = 5.96  ±  2.32 μM and IC50 = 7.36  ±  3.11 μM for BxPC-3 and MIA PaCa-2, respectively), while the YAPC and PANC-1 are more resistant to cisplatin action (IC50 = 56.7  ±  9.52 μM and IC50 = 100  ±  7.68 μM for YAPC and PANC-1, respectively).

Therefore, of these four lines, two are more sensitive and two are less sensitive to cisplatin. The subsequent studies were carried out on only two lines chosen between the most and least sensitive. We therefore chose the more sensitive BxPC-3 cell line and YAPC which, although not less sensitive than PANC-1, are however those in which fewer studies of this type have been so far carried out.

### 2.2. Cisplatin-Induced Apoptosis in BxPC-3 and YAPC Cells

**Figure 2:**
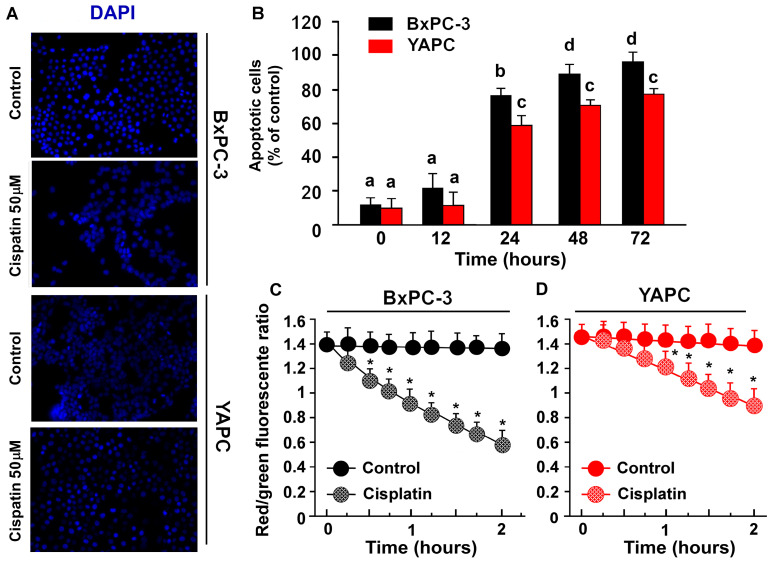
Analysis of mitochondrial membrane potential using the cationic dye JC-1 in BxPC-3 (**A**) and (**B**) YAPC cells. (**A**) Cells were incubated, or not, with 50 μM cisplatin for 24 hours and stained with 4′,6-diamidino-2-phenylindole (DAPI). The representative fields by confocal microscopy (magnification 40×) of one of four independent experiments are shown. (**B**) Quantification of the percentage of apoptotic nuclei was obtained using DAPI (means ± SD; n = 5). For both BxPC-3 and YAPC cells: *p* < 0.001 by one-way ANOVA followed by Bonferroni/Dunn post hoc tests; values with shared letters are not significantly different according to Bonferroni/Dunn. (**C**,**D**) Fluorescent spectra of JC-1 in BxPC-3 and YAPC cells treated or not with 50 μM cisplatin for the indicated time. The data are means ± S.D. of five different experiments and are presented as red J-aggregates/green monomer JC-1 fluorescence ratio. Asterisks indicate values that are significantly different (*p* < 0.05) from control at the same time point.

Mitochondrial membrane potential (Δψ_m_) is an important parameter of mitochondrial function, acting as an indicator of cell health [23]. Therefore, a decrement in Δψ_m_ accompanies early apoptosis in many systems. Thus, to evaluate the induction of apoptosis after cisplatin treatment in BxPC-3 and YAPC cells, we used the JC-1 probe to quantify the dissipation of Δψ_m_ since JC-1 possesses the ability to emit a signal at 590 nm, forming multimers known as J-aggregates following its accumulation in mitochondria at high membrane potential (red fluorescence) [24]. However, upon the depolarization of Δψ_m_, JC-1 switches to the monomeric state, emitting a signal at 529 nm (green fluorescence). The red/green fluorescence intensity ratio indicates the change in ΔΨ_m_ and then the occurrence of apoptosis. The shifts in fluorescence emission of JC-1, in BxPC-3 and YAPC cells treated, or not, with 50 μM cisplatin was then followed. ΔΨ_m_ remained high and stable for at least 2 h (red/green fluorescence ratio approximately equal to 1.4) in both control cell lines (i.e., cells incubated with the vehicle, without cisplatin). On the contrary, cisplatin caused a time-dependent decrease in ΔΨ_m_ which was much faster in BxPC-3 (significant already after 30 min of incubation) and much slower in YAPC (red/green fluorescence ratio values significantly different from the control after 90 min) (Figure 2C).

**Figure 3:**
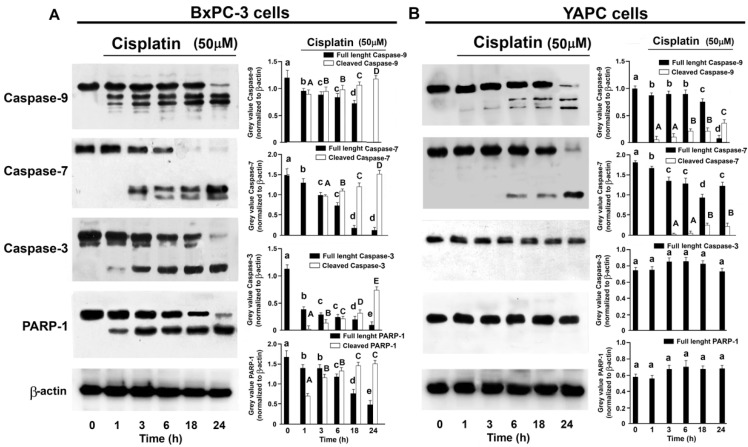
The cleavage of caspase-9, caspase-7, caspase-3 and Parp-1 induced by cisplatin in BxPC-3 (**A**) and YAPC (**B**) cells. Cells were treated with 50 µM cisplatin for the indicated time, and then subjected to Western blotting. Incubation with anti-β-actin confirmed the equal protein loading. The results shown are representative of five different experiments. The histograms on the right are representative of five independent experiments and the densitometry results are expressed as the mean ± SD (n = 5) of the sum of the gray level values of the westerns. (**A**) *p* < 0.001 by one-way ANOVA for all proteins. (**B**) *p* < 0.001 by one-way ANOVA for caspase-9 and -7; *p* > 0.05 by one-way ANOVA for caspase-3 and PARP-1. The values of histograms for full-length caspases and PARP with shared lower-case letters are not significantly different according to Bonferroni/Dunn post hoc tests. The values of histograms for cleaved caspases and PARP with shared capital case letters are not significantly different according to Bonferroni/Dunn post hoc tests.

To evaluate the cell death pathway activated in response to cisplatin, the proteolytic cleavages of caspase-3, -7, and -9, and PARP-1 were analyzed. As Figure 3A shows, when BxPC-3 cells were treated with cisplatin a sharp response was generated. In fact, the proteolytic cleavages of pro-caspases (Figure 3A) and PARP-1 (Figure 3A) demonstrate that these cells are indeed sensitive to the cytotoxic action of the cisplatin.

In YAPC cells treated with cisplatin, a decreased activation pattern of caspase 9 occurred, despite the appearance of the active fragment after 1h of treatment, the value of which remains constantly at low levels. Caspase 7 is also activated (Figure 3B) at a modest intensity and was, above all, significant after 18 h of treatment. Furthermore, caspase 3 (Figure 3B) and PARP-1 (Figure 3B) show no signs of activation, even 24 h after cisplatin treatment.

### 2.3. Cisplatin-Induced Autophagy in BxPC-3 and YAPC Cells

Experimental evidence demonstrates that autophagy promotes resistance of pancreatic cancer cells to treatment [25,26]. Therefore, we investigated whether the different response between BxPC-3 and YAPC cells to cisplatin was due to the activation of autophagy. Thus, we analyzed the conversion of LC3-I to LC3-II, the active form of LC3-I, essential autophagic markers in the process of elongation and maturation of phagophore.

**Figure 4:**
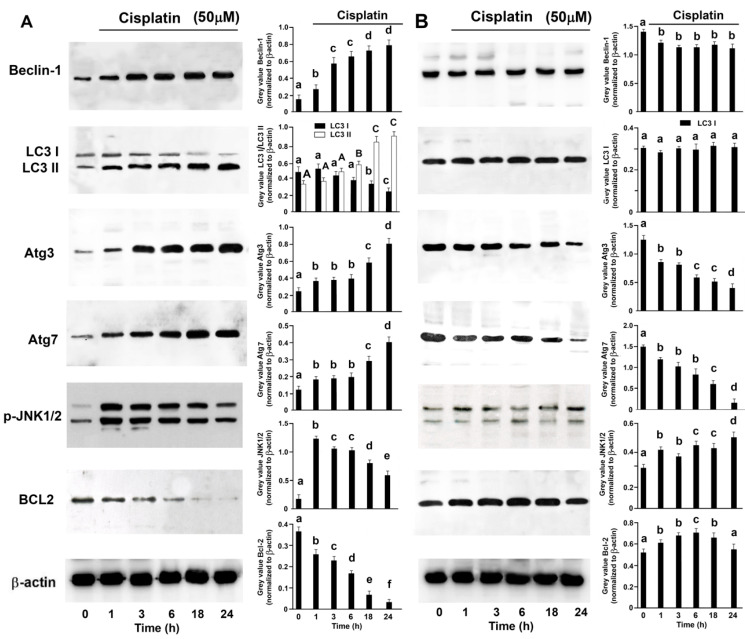
Cisplatin Induces Autophagy in BxPC-3 Cells. BxPC-3 (**A**) and YAPC (**B**) cells were treated with 50 µM cisplatin for different times. Cell lysates were analyzed using Western blotting, using specific antibodies. Sequential incubation with anti-β-actin confirmed the equal protein loading. Representative immunoblots of five experiments are depicted. The histograms on the right are representative of five independent experiments and the densitometry results are expressed as the mean ± SD (n = 5) of the sum of the gray level values of the westerns. (**A**) *p* < 0.001 by one-way ANOVA for all proteins. (**B**) *p* < 0.001 by one-way ANOVA for Atg3, Atg7, p-JNK1/2 and BCL2; *p* < 0.01 by one-way ANOVA for Beclin-1 and *p* > 0.05 by one-way ANOVA for LC3 I. The values of histograms with shared lower-case letters are not significantly different according to Bonferroni/Dunn post hoc tests. The values of histograms for LC3 II with shared capital case letters are not significantly different according to Bonferroni/Dunn post hoc tests.

50 µM cisplatin induced LC3-I (19 kDa) to LC3-II (17 kDa) conversion in a time-dependent manner in BxPC-3 cells (Figure 4A), whilst no effects were seen in YAPC cells (Figure 4B). During the autophagosome formation, Beclin-1, after detachment from Bcl-2, forms a complex with Vps34 facilitating the recruitment of Atg proteins. It is known that c-Jun N-terminal kinase (JNK1/2) activation leads to Bcl-2 phosphorylation and Beclin-1 detachment [27]. Indeed, in BxPC-3 cells treated with cisplatin, Bcl-2 decreased, Beclin-1, Atg-3 and Atg-5 increased, and, finally, the phosphorylation of JNK1/2 also increased significantly (Figure 4A).

### 2.4. The Involvement of ERK1/2 Activation in the Cisplatin Response of YAPC Cells

**Figure 5:**
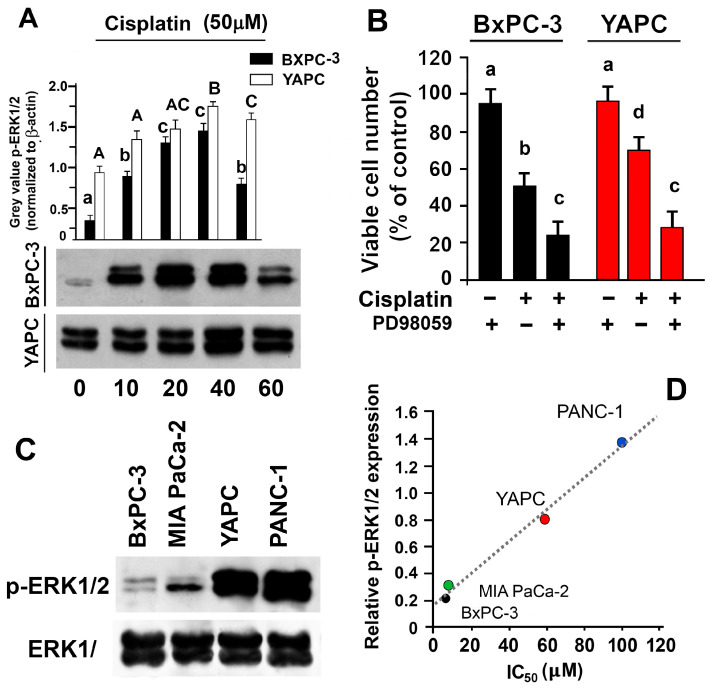
The effects of cisplatin on ERK1/2 activation. (**A**) BxPC-3 and YAPC cells were treated with 50 µM cisplatin for different times and cell lysates were analyzed by Western blotting, using activated ERK1/2 specific antibody. (**B**) Cells were pre-treated for 45 min with 25 µM PD98059 and then incubated or not with 50 µM cisplatin for 24 h. Then, cell viability was measured with sulforhodamine B colorimetric assay. (**C**) Basal expression levels of ERK1/2 in cell lines. Cell lysates were analyzed using Western blotting, using anti-phospho-ERK1/2 (p-ERK1/2) or anti-total ERK1/2 antibodies. (**D**) The relationship between the levels of phosphorylated ERK1/2 and the IC_50_ of cisplatin in the pancreatic cell lines used. Sequential incubation with anti-β-actin confirmed the equal protein loading. Representative immunoblots of five experiments are depicted. The histograms on the right are representative of five independent experiments and the densitometry results are expressed as the mean ± SD (n = 5) of the sum of the gray level values of the westerns. A and B, *p* < 0.001 by one-way ANOVA followed by Bonferroni/Dunn post hoc tests. Values with shared letters are not significantly different according to Bonferroni/Dunn. The lower-case letters refer to BxPC-3 cells and capital case letters to YAPC cells.

After having observed the involvement of the JNK1/2 MAPK in autophagic control, we checked whether another MAPK, ERK1/2, with a greater anti-apoptotic vocation, was also involved in the response to cisplatin, as evidenced in other cells [28,29,30,31]. Figure 5A shows that ERK1/2 was activated in both cells treated with 50 µM cisplatin. Thus, 25 μM PD98059 (inhibitor of the ERK upstream kinase MEK) was used to inhibit ERK1/2 in BxPC-3 and YAPC cells treated with 50 μM of cisplatin. The results show that both BxPC-3 and YAPC cells were significantly more sensitive to cisplatin-mediated apoptosis compared to cells exposed to cisplatin alone (*p* < 0.05; Figure 5B). These findings argue that ERK1/2 inactivation plays a significant functional role in the potentiation of cisplatin lethality. Notably, levels of activated ERK1/2 (p-ERK1/2) in cells in the absence of cisplatin are different in each cell line (Figure 5C), and cisplatin IC50 values are positively correlated with such levels in pancreatic cell lines (Figure 5D).

## 3. Discussion

Pancreatic ductal adenocarcinoma (PDAC), the most common type of pancreatic cancer, is one of the most lethal neoplasms, with an increasing incidence [1,2]. Its high mortality rate is often related to late diagnosis, high invasiveness, and resistance to treatments [6,15]. Over the past two decades, the most used treatment for PDAC has been based on gemcitabine and, more recently, on its combination with the agent erlotinib or albumin-bound paclitaxel [32]. Another four-drug combination, including the oxaliplatin together with irinotecan, fluorouracil, and leucovorin (Folfirinox), showed a modest improvement in response, overall survival, and progression-free survival compared to gemcitabine as monotherapy [33]. Cisplatin, as a single agent or as an adjunct to combination chemotherapy, is also being evaluated for early, advanced, or metastatic PDAC in several ongoing clinical trials [18,34]. The cisplatin cytotoxicity is regulated by the Fanconi anemia/BRCA pathway [35], which is downregulated in numerous pancreatic cancers [36]; therefore, the use of cisplatin in the treatment of PDAC makes reasonable sense as these tumors may be more sensitive. However, as the clinical utility of cisplatin is limited for acquired resistance phenomena [18], understanding the mechanisms involved in PDAC cell resistance could help to refine the use of cisplatin in pancreatic cancer chemotherapy.

This study investigated the molecular mechanisms underlying the sensitivity and resistance to cisplatin in different pancreatic ductal adenocarcinoma cell lines, aiming to identify potential therapeutic targets that could enhance treatment efficacy and overcome chemoresistance, a major clinical challenge in managing this highly lethal cancer.

Thus, we examined the response to cisplatin treatment in four pancreatic cancer lines (BxPC-3, Mia Paca-2, PANC-1, YAPC) with different genotypic and phenotypic characteristics [37,38] and the molecular mechanisms associated with their acquired resistance. These considerably different PDAC cell lines were selected to represent the diversity that can be encountered in this tumor type and the development of its resistance to cisplatin. Since cisplatin is one of the most effective inducers of apoptosis in pancreatic cancer cells [39,40,41], it proved to be effectively cytotoxic in the BxPC-3 and Mia Paca-2 pancreatic lines. In contrast, the YAPC and PANC-1 lines demonstrated remarkable resistance. Thus, our results confirm the heterogeneity of PDAC cells in response to cisplatin; by exploiting heterogeneity, based on cisplatin sensitivity, we effectively selected suitable cell lines for our studies on PDAC chemoresistance to cisplatin. The characteristic responses to cisplatin in BxPC-3 and YAPC cell lines were compared, including the differential activation of molecular pathways, to better understand the nature of PDAC chemoresistance.

Regarding the mechanisms involved in the resistance of BxPC-3 cells to chemotherapy, several important molecular targets and pathways have been elucidated in detail to date [42,43,44,45,46]; however, the exact mechanism of action is not fully understood, and the intrinsic cell complexity, at the basis of molecular individuality of the pancreatic [47] supports the implementation of further studies. Concerning drug-resistant Panc-1 and YAPC cell lines, previous studies have clarified some mechanisms related to the chemoresistance of PANC-1 cells [48,49,50], but nothing is known about YAPC cells.

As far as gemcitabine is concerned, some studies indicated the importance of the autophagic process in the acquisition of resistance in PDAC [51,52,53], and autophagy is necessary for pancreatic cancer growth [26,54]. Congruently, Fujii et al. [55] demonstrated that autophagy was activated in pancreatic tumor tissue and that it negatively correlated with resistance to therapy. In addition, PANC-1 cells in basal conditions showed activated autophagy. In both PANC-1 and BxPC-3 cells, autophagy significantly increases after treatment with 5-fluorouracil or gemcitabine, having a protective effect against anticancer drugs. Since YAPC cells have a greater ability to survive cisplatin treatments, whilst BxPC-3s are more sensitive, we monitored the autophagic markers Beclin-1 and LC3 I/II. Firstly, the basal levels of LC3 I expressions were different in the two lines, and it is known that they can vary considerably between different cell types and in response to different stresses. Secondly, in BxPC-3 cells treated with cisplatin we detected high levels of LC3 II. During autophagy, LC3 I is conjugated with phosphatidylethanolamine to form LC3 II and stably associated with the autophagosome membrane; therefore, LC3 II is widely used as an autophagic biomarker [56]. Thirdly, in YAPC the maturation of LC3 I/II is not observed. All this is confirmed by evaluating the modulation of Atg 3 and Atg 7 (two proteins that have a key role in the maturation process of LC3 I/II) in these cell lines; in YAPC cells, unlike BxPC-3, Atg 3 and Atg 7 expressions decreased. Bcl-2 inhibits autophagy by binding to and impeding Beclin-1, an autophagy promoting protein [56]. Our study revealed that cisplatin in BxPC-3, but not in YAPC cells, suppresses the expression of Bcl-2 and increases the expression of Beclin-1, thus activating autophagy. Moreover, Bcl-2 is a tumor suppressor able to inhibit apoptosis and promote cell survival. Thus, the inhibition of Bcl-2 in BxPC-3 cells could also explain why cisplatin is able to induce apoptosis.

Activation of the JNK pathway results in Bcl-2 phosphorylation, an event known to enhance autophagy by disrupting the Bcl-2/Beclin 1 competitive interaction [57]. Similarly, we observed that JNK1/2 was phosphorylated in cisplatin-treated BxPC-3, cells but not in YAPC cells. Our current data suggest that autophagy is strongly induced as a defense mechanism against cisplatin in BxPC-3, that is well in line with previous observations, but not in PANC-1 cells. The different sensitivity to cisplatin of BxPC-3 and PANC-1 cells is probably due to intrinsic differences in the specific characteristics of the two tumor cell lines, and in the role of differentially expressed genes in the resistance of cancer cells to cisplatin [38]. For example, Chadha et al. [58] reported overexpressed activated ERK1/2 in pancreatic carcinoma. In vitro and in vivo studies indicated that ERK1/2 plays a prominent role in gemcitabine resistance within pancreatic cancer cells. Most importantly, it was demonstrated that ERK1/2 blockage enhanced gemcitabine’s chemotherapeutic potential in vitro among pancreatic cancer cells [59,60]. Here, based on the direct association observed between basal levels of activated ERK1/2 and cisplatin sensitivity in different cell lines, we hypothesize that such levels of activated ERK1/2 may be related to different degrees of differentiation, tumorigenicity and cisplatin resistance of these cells.

Furthermore, KRAS mutations, such as the KRASG12D mutation found in Panc-1 cells, are known to activate several downstream signaling pathways, including the ERK1/2 pathway. The constitutive activation of ERK1/2 signaling in KRAS-mutant cells leads to increased cell survival and metabolic adaptations, which help the cells evade the cytotoxic effects of chemotherapy [21]. Moreover, the sustained activation of this pathway can lead to feedback mechanisms that further enhance the resistance to chemotherapy by activating compensatory survival pathways [22]. These findings suggest that the enhanced cisplatin resistance observed in Panc-1 cells could be driven by such adaptive survival mechanisms mediated by ERK1/2 signaling.

In conclusion, we have given some molecular indications capable of partially explaining the different effects of cisplatin in the two pancreatic tumor cell lines and related to the apoptotic and autophagic machinery as well as a possible role of the MAPKs.

**Figure 6:**
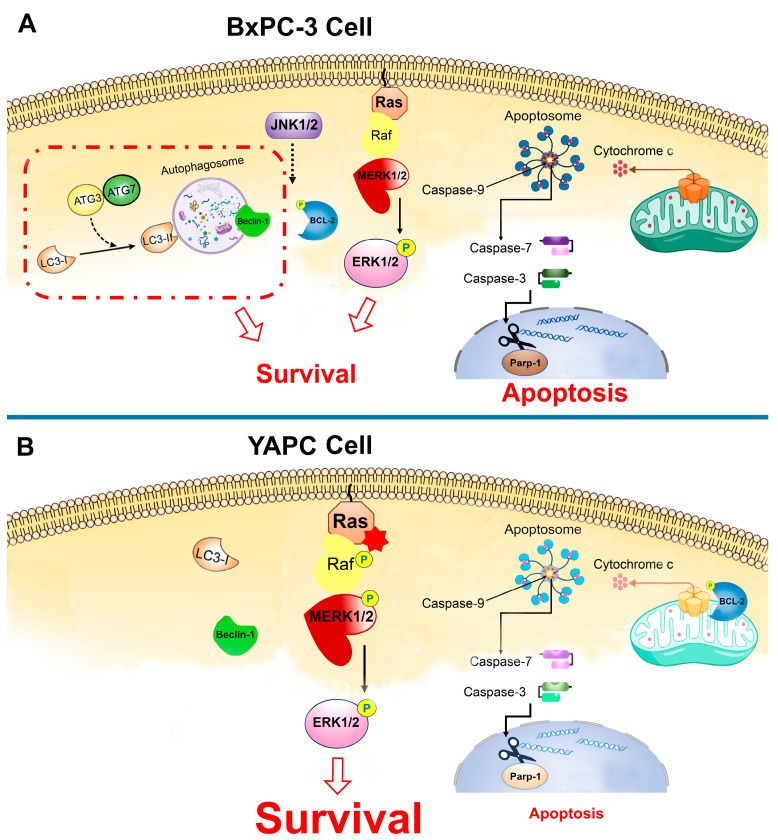
The schematic diagram illustrates the intracellular signaling mechanisms in pancreatic cancer cells. (**A**) BxPC-3 cells, in response to cisplatin, activate key pathways such as apoptosis (pro-caspase-9, -7, -3 cleavage), as well as autophagy (LC3-I to LC3-II conversion) and ERK1/2 signaling, promoting survival. (**B**) YAPC cells with a KRAS mutation exhibit constitutive ERK1/2 pathway activation; consequently, there is a decreased activation pattern of caspase-9, caspase-7, and caspase-3, along with PARP-1 cleavage, representing apoptosis resistance. Additionally, YAPC cells do not show autophagy activation.

We have summarized these differences in mechanisms of action in a schematic diagram (Figure 6).

Future research directions may also be highlighted, exploring the therapeutic potential of targeting autophagy and MAPK pathways to overcome resistance, and investigating novel biomarkers for predicting cisplatin sensitivity in diverse pancreatic cancer subtypes.

## 4. Materials and Methods

### 4.1. Cell Cultures

YAPC (DSMZ, Braunschweig, Germany) cells were cultured in RPMI 1640 medium (EuroClone, Pero, MI, USA) supplemented with 10% (vol/vol) heat-inactivated fetal bovine serum (FBS), glutamine 2 mM, penicillin (100 U/mL), and streptomycin (100 mg/mL). BxPC-3, MIA PaCa-2, and PANC-1 cells (ATCC, Rockville, MD, USA) were cultured in Dulbecco’s Modified Eagle Medium (DMEM) (4.5 mg/L glucose) (EuroClone, Pero, MI, USA) supplemented with 10% (vol/vol) heat-inactivated FBS, glutamine 2 mM, penicillin (100 U/mL), and streptomycin (100 mg/mL). Cells were grown in a humidified incubator containing 5% CO_2_ in air at 37 °C and used for biological assays when 70–80% confluence was reached.

### 4.2. Cell Viability Assay

Pancreatic cancer cell viability was measured with sulforhodamine B (SRB) colorimetric assay, as previously shown [61]. Viable cells were also counted by the trypan blue exclusion assay and light microscopy. The data presented are means ± standard deviation (SD) from eight replicate wells per microtiter plate.

### 4.3. Preparation of Subcellular Fractions and Western Blotting

Subcellular fractions were obtained as previously reported [62]. Western blotting analysis, immunodetection, and densitometric analysis were performed as previously described [63].

### 4.4. Spectroscopic Analysis of Mitochondrial Membrane Depolarization

Mitochondrial membrane depolarization was monitored by fluorescent spectrophotometer as previously reported [64].

### 4.5. Data Analysis

Results are shown as means ± SD. Statistical analysis was carried out using ANOVA and as indicated, post hoc tests (Bonferroni-Dunn) were also performed. A *p* value less than 0.05 was considered to achieve statistical significance.

## Data Availability

Dataset available on request from the authors.

## Supplementary Materials

The following supporting information can be downloaded at: https://www.mdpi.com/article/10.3390/ijms252413662/s1.
