# Supplementary material for: Different Cytotoxic Effects of Cisplatin on Pancreatic Ductal Adenocarcinoma Cell Lines
Source: Int J Mol Sci. 2024 Dec 20;25(24):13662. doi: 10.3390/ijms252413662 (PMC11727771; doi:10.3390/ijms252413662)
Supplement: Supplementary file 1 [file ijms-25-13662-s001.zip › ijms-3355274-supplementary.pdf]

Supplementary Table S1. IC50 Values of Cisplatin in Pancreatic Tumor Cell Lines

	IC50 (µM)		
**Cell Line**	**24 hours**	**48 hours**	**72 hours**
**BxPC-3**	26.1 ± 3.9	5.9 ± 2.3	3.7 ± 3.3
**MIA PaCa-2**	29.4 ± 2.7	7.36 ± 3.1	3.42 ± 3.1
**PANC-1**	>200	100 ± 7.7	51.2 ± 3.8
**YAPC**	133.7 ± 2.6	56.7 ± 9.5	9.38 ± 2.9
